# Biomechanical evaluation of an arthroscopic transosseous repair as a revision option for failed rotator cuff surgery

**DOI:** 10.1186/s12891-018-2089-4

**Published:** 2018-07-19

**Authors:** Felix Dyrna, Andreas Voss, Leo Pauzenberger, Elifho Obopilwe, Augustus D. Mazzocca, Alessandro Castagna, Cory Edgar

**Affiliations:** 1 Department of Orthopaedic Sports Medicine, Technical University, Munich, Germany; 2 Department of Orthopaedic Surgery, University of Connecticut, Farmington, CT USA; 3 Department of Shoulder and Elbow IRCCS Humanitas Institute, Milan, Italy

**Keywords:** Rotator cuff, Cuff repair, Revision, Transosseous repair, Arthroscopic repair

## Abstract

**Background:**

The number of revision rotator cuff cases is increasing. The literature is lacking guidance or biomechanical evaluation for fixation strength in a revision case scenario. Therefore, the aim of the study was to provide biomechanical data investigating primary fixation strength of a transosseous technique after anchor pullout failure of a single row reconstruction. It was hypothesized that an arthroscopic transosseous repair system as a procedure for rotator cuff revisions is providing equivalent stability compared to a primary single row suture anchor fixation due to change of fixation site.

**Methods:**

Eight matched pairs (*n* = 16) of fresh frozen human shoulders were tested. The paired specimen shoulders were randomly divided into two repair groups (**A** single row and **B** primary transosseous repair). The potted specimens were mounted onto the Servohydraulic test system. Both groups were tested under cyclic loading followed by load to failure testing. Suture anchor repair shoulders (group A) that were tested to failure underwent a revision transosseous repair and were subsequently tested again using the same setup and protocol (group C).

**Results:**

The mean native footprint areas did not show a significant difference between groups. The reconstructed footprint area showed a significantly greater coverage in favor of the transosseous repair. Ultimate load to failure of reconstructions with the primary anchor fixation (344.73 N ± 63.19) and the primary transosseous device (375.36 N ± 70.27) was not significantly higher compared to the revision repair (332.19 N ± 119.01 *p* = 0.45, *p* = 0.53).

**Conclusion:**

The tested transosseous anchor device is a suitable option to widely used suture anchors, providing equivalent fixation properties even in a revision case scenario.

**Level of evidence:**

Basic Science Study, Biomechanics.

## Background

Despite promising results in biomechanical studies, the clinical re-tear rate of rotator cuff repairs is still high. In the literature the incidence of recurrences has been reported of over 30% for small injuries [1, 2] and may extend to 90% in cases of massive tears [3–6]. Reasons for failure of reconstruction are variegated [7]. Rotator cuff revision surgery is always challenging and results are often inferior to primary repairs [8, 9]. Arthroscopic repair may present advantages compared to historical open transosseous repairs, such as minimal infraction of the deltoid muscle, the ability to dynamically inspect the entire glenohumeral joint for associated pathologies, characterize tear patterns, and decrease postoperative pain and stiffness [10, 11].

The quest for improved methods of rotator cuff repair led to the development of all-arthroscopic transosseous rotator cuff repair techniques [12], which could potentially combine the biomechanical strengths of open transosseous repairs with all of the advantages of arthroscopic techniques. Recent studies, that have compared traditional transosseous equivalent techniques with modern single- and double-row anchor-based configurations, showed superior load to failure strength in transosseous repairs [13, 14].

The purpose of the present study was to compare displacement under cyclic loading, load-to-failure, and footprint coverage of rotator cuff reconstructions performed with a new, all-arthroscopic transosseous repair (TOR) system and an established single row suture anchor repair configuration, as (a) a primary repair technique and (b) in case of failed anchor based initial repair. Our primary hypothesis was that an arthroscopic transosseous repair system as a procedure for rotator cuff revisions is providing equivalent stability compared to a primary single row suture anchor fixation due to change of fixation site.

## Methods

### Preparation

The test involved the use of eight matched pairs (*n* = 16) fresh frozen human shoulders without macroscopic evidence of rotator cuff pathology. The mean age of specimens was 69,9 years with a range from 74 to 66 years including 8 female and 8 male specimens. All specimens were obtained from Medcure Inc. (Portland, OR). The study was reported via Human Research Determination Form to the institutional review board (IRB) of the University of Connecticut and it was documented, that no IRB approval was required (de-identified specimen do not constitute human subjects research). Specimens were thawed for 24 h at room temperature prior to dissection. Bone Density via micro-CT (Lunar DXE, Madison, Wis) was performed to ensure consistent bone quality among tested specimens at the greater tuberosity in a consistent manner at a 1 × 1-cm area.

Selected specimens were prepared by removing all soft tissues, but leaving the supraspinatus, infraspinatus, and teres minor tendons intact. The native supraspinatus footprint was measured with digital calipers for linear measurements (Absolute Digimatic; Mitutoyo, Kawasaki, Japan). While the supraspinatus muscle was held in a superior direction, the width of the footprint at its greatest dimension was measured by placing 1 limb of the digital caliper precisely on the articular edge of the intact supraspinatus tendon and the other arm of the caliper on the lateral bursal-side edge of the insertion. The anterior and posterior insertions of the supraspinatus were measured likewise. This procedure allowed an estimated contact area to be calculated. Although the supraspinatus footprint does not represent a true rectangle, these measurements provided a consistent means to compare the contact areas of the intact and repaired supraspinatus tendons. To ensure correct measurements, results were compared with a measured area using a MicroScribe digitizer (Immersion, San Jose, CA) by lifting the supraspinatus muscle and tracing the outline of the attachment [15], before creating the tear. The same investigator performed all footprint measurements. The next step involved the sharp dissection of the supraspinatus tendon from its footprint to create a uniform 20x10mm full-thickness tendon tissue defect approximately 5 mm proximally to the humeral insertion of the tendon, as previously described [16].

Prior to the repair the footprint area was macroscopically completely decorticated to mimic a revision case scenario and weaken the bone quality. The paired specimen shoulders were randomly divided into two groups. A total of 8 specimens per group were tested.

### Repair configurations

#### Group **A** Single Row

**Fig. 1:**
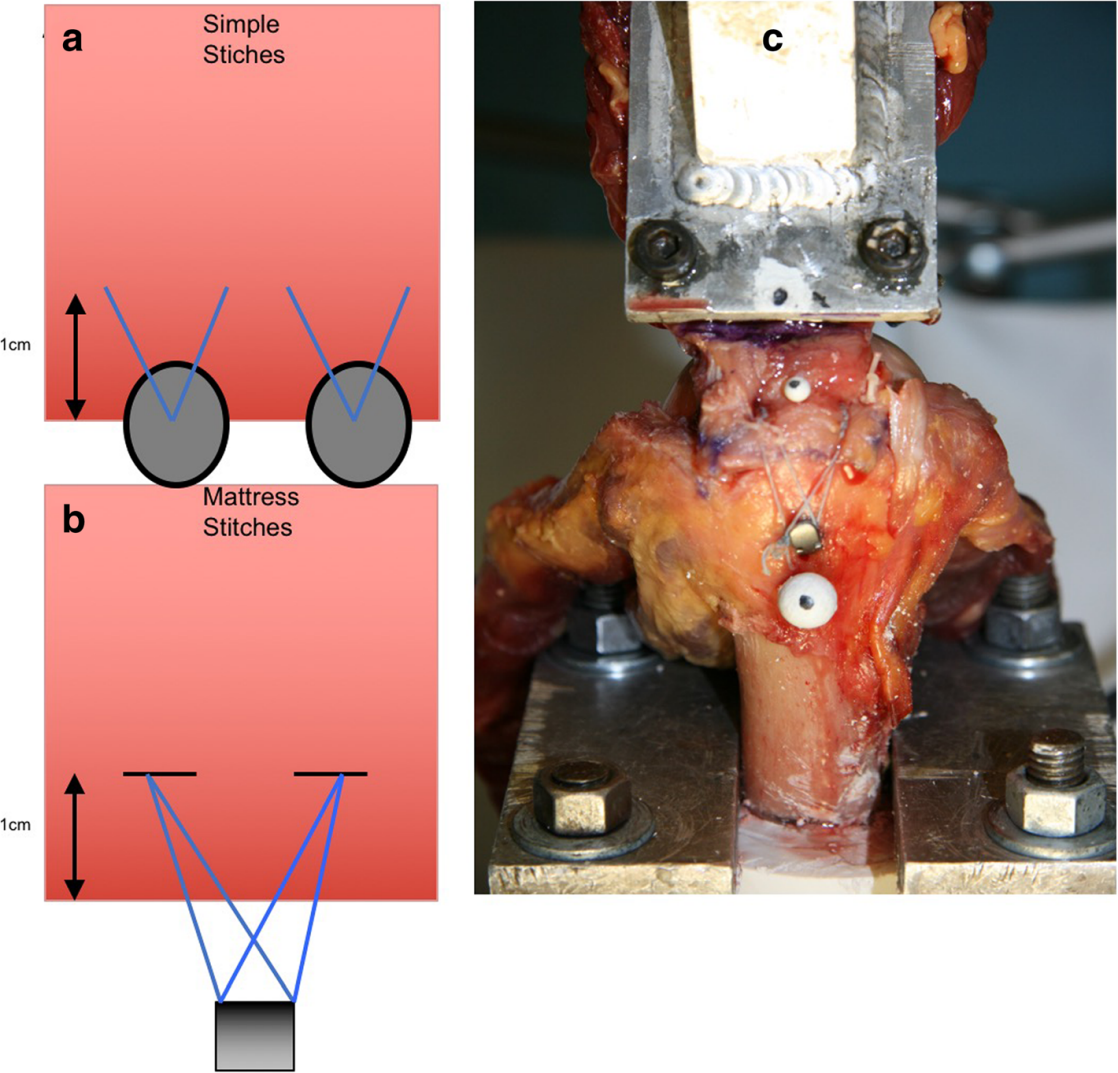
shows (**a**) single row repair, (**b**) TOR with Sharc-FT, (**c**) TOR repair final set-up

The single-row repair was performed using two double loaded titanium corkscrew anchors (Arthrex, Naples, FL) placed lateral to the cartilage margin in the greater tuberosity 12 mm apart. The anchors were placed at the “dead man’s angle” to maximize pullout strength [17]. Both strands of No. 2 FiberWire (Arthrex, Naples, FL) in each anchor were then shuttled in a typical fashion using a Suture Lasso (Fig. 1a). Knot tying was performed in arthroscopic fashion using a standard knot pusher with overhand throws, alternating half hitches and posts to maximize loop and knot security [18].

#### Group **B** primary TOR

The transosseous repair was performed with the Sharc-FT® system (NCS Lab, Medical Devices Factory), which is based on diverging canals created by a drill guide the Taylor stitcher® (NCS Lab, Medical Devices Factory) through the great tuberosity of the humerus consisting of one lateral entering and - depending on tear size - multiple medial exit tunnels. The transosseous configuration involved the creation of a lateral entry hole centered laterally from the lesion and two medial exit holes, one anterior and one posterior with a distance of 10-12 mm in between. Therefore, the first medial exit tunnel, a 2 mm hole in the ventral third of the footprint was punched with the guidance of the Taylor stitcher® device (NCS Lab, Medical Devices Factory). Furthermore, this guide was used to place the lateral 2 mm hole 3 cm from the greater tuberosity edge. The posterior medial exit tunnel was created in the same manner while keeping the guide in the lateral exit hole. Once the canals were placed, the Sharc-FT (NCS Lab, Medical Devices Factory) device preloaded with two internal sutures was used to shuttle the definite sutures, No. 2 FiberWire (Arthrex, Naples, FL). These sutures were arranged to obtain two mattress stitches over the tendon (Fig. 1b), then the four suture limbs were used to close the suture loop passing them through the eyelet on the device to function as a second row increasing the footprint coverage and area of compression [19].

#### Group **C** revision Transosseous repair with Sharc-FT

After load-to-failure testing of the single-row group, shoulders, which showed a sufficiently intact footprint and tendon after initial testing, underwent a revision procedure with the Sharc-FT(R) (NCS Lab, Medical Devices Factory) system following the before mentioned technique. Hence, comparing transosseous refixation with this device as a suitable revision technique. Again the aiming device (Taylor stitcher) was used to place as previously descripted the two converging tunnels at the spot of the removed anchors and the revision repair was completed.

### Biomechanical testing

All samples were tested in air at room temperature. The humerus was cut 20 cm from the joint, centered in a polyvinylchloride pipe, and potted with plaster of Paris. The potted cadaveric specimen was then mounted on the testing apparatus with the tendons oriented in line with the anatomic pull of the myo-tendinous unit The medial portion of the supraspinatus tendon was freeze-clamped and attached to a MTS, Eden Prairie, MN) as described in previous studies [18, 20]. In this setup, the specimens were tested under cyclic load, while measuring gap formation. To quantify gap formation one marker was placed laterally below the greater tuberosity and one in line centric on the musculotendinous junction of the supraspinatus.

A video digitizing system that involved video recording and computer digitization of the markers, creation of centroids representing the center of the markers, and the calculation of distances between them with MaxTRAQ 2D (Innovision Systems, Inc., Columbiaville, Michigan) software was used (Fig. 1c).

After a preload phase with 10 N for 1 min and ten initial cycles, the load was set to change from a minimum value of 10 N to a maximum of 180 N at a velocity of 33 mm/s [21–24] for a total of 200 cycles [21, 25–27]. In the event of gap formation greater than 5 mm, the test was stopped and the number of completed cycles was noted. After a preload phase samples that reached 200 cycles with a gap variation less than 5 mm were then tested to load-of-failure at a rate of 33 mm/s.

Shoulders from the anchor repair group A that were tested to failure underwent a revision transosseous repair and were subsequently tested regarding gap formation under cyclic loading and load-to-failure using the same setup and protocol group C.

### Statistical analyses

Given the exploratory nature of this study, an a priori power analysis was difficult. We anticipated a medium effect size (30–45% difference in load to failure or about 100 N) between the primary suture anchor and revision transosseous repair with an estimated variability of ±50 N. A sample size of 6 per group will provide 80% power to detect a difference of 100 N an alpha value of 0.02 between the repair groups. Descriptive statistics were calculated using mean and standard deviation for the repair groups. Both independent and pair t test were used to compare differences in load to failure, slope, displacement, and footprint reconstruction. The revision trans-osseous repair group included the same shoulders tested in primary suture group creating dependency within the data which necessitated the use of separate t tests as opposes to a global ANOVA. To avoid inflating our Type I error rate, a Bonferroni adjusted *p* value of 0.02 was used as the threshold for statistical significance. All statistical analysis was performed using Stata 12 (StataCorp. 2011. *Stata Statistical Software: Release 12*. College Station, TX: StataCorp LP.).

## Results

**Fig. 2:**
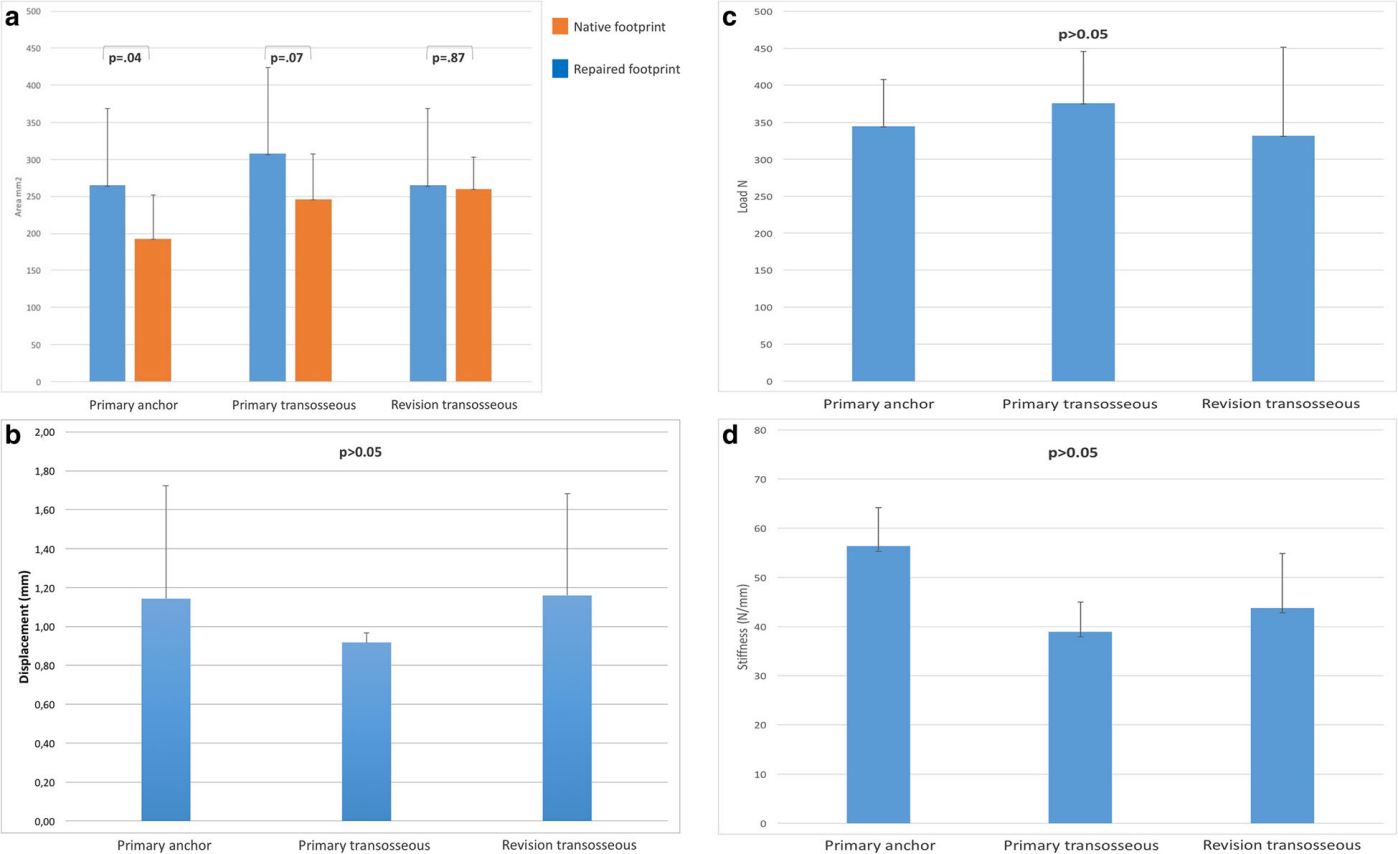
(**a**) displaces the native and repaired footprint data for each repair technique, (**b**) represents displacement data after cyclic loading, (**c, d**) shows ultimate load to failure results and stiffness of the repair constructs

The results for footprint reconstruction, displacement during cyclic loading, load to failure and stiffness were summarized in Fig. 2 a-d. The bone quality (BMD) was measured for each specimen and did not differ between groups (group A mean 0.361 g/cm^2^ ± 0.05 and group B 0.435 g/cm^2^ ± 0.09 *p* = 0.094).

### Footprint coverage

The mean native footprint areas in group **A** 264.91 mm^2^ ± 103.12, and group **B** 307.68 mm^2^ ± 116.13 did not show a significant difference *p* = 0.48. Since group **C** includes the same specimens as group **A** the native footprints are equal. The covered footprint areas after the repair in group **A** 192.48 mm^2^ ± 59.55, group **B** 246.25 mm^2^ ± 61.16, and group **C** 259.93 mm^2^ ± 43.28 were compared A vs. B *p* = 0.01 and A vs. C *p* = 0.12 in favor of the transosseous repair.

### Displacement

200 cycles of repetitive motion at time zero resulted in no significant differences between the three groups (A vs. B *p* = 0.57, A vs. C *p* = 0.98 and B vs. C *p* = 0.47). Mean displacement between the groups was not significant and represented in Fig. 2b An overall displacement over 5 mm was considered as a failure. This occurred once in group **A** and group **B** as the anchor already pulled out but not in group **C**. (Fig. 2b).

### Ultimate Load to Failure

Ultimate load to failure of reconstructions with the primary anchor fixation (344.73 N ± 63.19 group A) and the primary Sharc-FT device (375.36 N ± 70.27 group B) was not significantly higher compared to the revision repair (332.19 N ± 119.01 group C, A vs. B *p* = 0.45, A vs. C *p* = 0.53). There were also no significant differences comparing the slope of the constructs, and yield loads. (Fig. 2c, d). The failure modes were 5/8 (63%) anchor pullouts and 3/8 (37%) suture pull through in group A. For group B the main failure was suture cutting through tendon 5/8 (63%) and 3/8 (37%) bony suture pullouts. Group C showed 6/8 (75%) anchor pullouts and 2/8 (25%) suture tendon interface failures.

## Discussion

The purpose of this study was to evaluate the load to failure, stiffness, displacement under cyclic loading, and footprint reconstruction of two different fixations at time zero. The number of suture passages through the tendon was equal (*n* = 4) between the groups to focus on bony fixation strength. Generated results did confirm the original hypothesis that the transosseous fixation technique restores similar fixation strength in a revision case scenario compared to primary single row anchor fixation. We found no significant difference in the biomechanical properties of the compared techniques. The rotator cuff repair represents one of the most frequently performed orthopedic surgical intervention [28]. Hence of numerous technological innovations, the re-tear rate is still unsatisfactory high [3, 4, 7, 10] .With a raising number of surgeons performing their arthroscopic rotator cuff repairs with suture anchors our attention has been placed on a possible revision fixation technique. To our mind the TOR serves as a reasonable option to fulfill this task preserving similar biomechanical parameters by changing the fixation site. McLaughlin was the first one describing a transosseous rotator cuff repair back in 1944 [29]. Since then a progress of technological improvements established a save and reproducible technique that can even be performed arthroscopically [19]. According to Gerber et al. [30], in theory a perfect repair should combined high fixation strength, minimal gap formation, and sufficient mechanical stability for tendon-bone healing. The existing literature consist of studies demonstrating that transosseous techniques have equivalent biomechanical properties relative to suture anchor fixation [21, 31–33]. Lately, Behrens et al. [34] showed that traditional transosseous suture construct without anchors can even provide an initial fixation strength equivalent to suture bridge repairs. The stress load is distributed wider in the bone tunnel of the transosseous repair, whereas in suture anchor repair, resulting in less local stress loads [35]. In addition, the pressure is more homogenously assigned in transosseous sutures in opposite to the anchor repair, where high values may be a risk factor for ischemic tissue damage [36]. To evaluate the Gap-formation and displacement two optical markers were positioned centric on either side of the repair as shown in Fig. 1c. This position was chosen to analyze the average displacement since Tashjian et al. [32] found that gapping significantly increased from the anterior to posterior region. The gap formation observed in our three groups did not differ significantly and approached similar values as previously reported in literature [34]. Hence, we had two failures in groups A and B and no failure in group C during cyclic loading due to anchor pull outs caused by weak bone quality. The bone quality (BMD) was measured for each specimen and did not differ between groups (group A mean 0.361 g/cm^2^ ± 0.05 and group B 0.435 g/cm^2^ ± 0.09 *p* = 0.094). The macroscopically decortication was performed after the BMD measuring and prior to the repairs to mimic poor bone quality. In regarding to Tingart et al. [37] who published that a successful rotator cuff repair highly depends the bone density at the greater tuberosity we expected to weaken our primary suture anchor repair by this decortication process but we still reached comparable anchor pull out values compared to the literature [31]. Pointing out that we may did not weaken the bone significantly even if macroscopically a full removal of cortical bone was performed. Pointing out that the suture anchors mainly rely on the trabecular, not the cortical bone [38]. During our ultimate load to failure testing, anchor pullout was the main failure mode in all groups A-C. The most interesting factor is that we were able to show that even after pulling out a suture anchor repair the subsequent implanted TOR was not significantly weaker than the initial anchor nor the initial TOR. Leading to the assumption that after a failed single row anchor repair the TOR is a possible revision option.

Knowing the initial strength characteristics of different rotator cuff repair techniques may influence surgical decision-making and may ultimately improve patient outcomes. Traditionally, the TOR technique was performed open and later modified to mini-open procedures. Apreleva et al. [39] demonstrated that TOR provides superior supraspinatus footprint coverage compared to suture anchor systems. Similar results were found in our study comparing a transosseous repair with a single row suture anchor.. The primary limitation of this study is that it is an in-vitro study only incorporating fixation strength at time point zero and a small sample size. The results cannot be extrapolated to the potential impact of tendon healing. Further the study specimens had intact rotator cuff tendons that do not replicate normal tendon and joint degeneration. We also created an isolated supraspinatus defect, which does not necessarily replicate the clinical situation. We try to include specimens with comparable age and bone quality. Lastly, the repairs in our study were performed under the ideal condition of being open procedures. Finally, the results of our study suggest that the initial fixation strength of single row suture anchor and TOR for rotator cuff repair at time point zero with comparable tendon and bone is equivalent. Both reconstructions prevented Gap-formation and early failure but preserved good footprint coverage. The revision TOR proved its biomechanical properties to be fully capable as an option for primary and secondary repairs. The clinical relevance of this study showed that there is no difference when comparing a current used arthroscopic single row suture anchor repair with a novel arthroscopic transosseous technique, supporting its use as a revision option.

## Conclusion

The tested transosseous anchor device is a suitable option to widely used suture anchors, providing equivalent fixation properties even in a revision case scenario for single row reconstructions.

## Abbreviations

ANOVA: Analysis of variance
BMD: Bone mineral density
TOR: Transosseous repair
